# Impact of Concentration Polarization Phenomena on Gas Separation Processes with High-Performance Zeolite Membranes: Experiments vs. Simulations

**DOI:** 10.3390/membranes14020041

**Published:** 2024-02-01

**Authors:** Omar Abdul Majid, Margarita Kuznetsova, Christophe Castel, Eric Favre, Rainier Hreiz

**Affiliations:** Université de Lorraine, CNRS, LRGP, F-54000 Nancy, Francechristophe.castel@univ-lorraine.fr (C.C.); rainier.hreiz@univ-lorraine.fr (R.H.)

**Keywords:** gas, separation, process, polarization, zeolite, simulation

## Abstract

Polarization phenomena play a key role in membrane separation processes but remain largely unexplored for gas separations, where the mass transfer resistance is most often limited to the membrane. This assumption, which is commonly used today for the simulation of membrane gas separations, has to be reconsidered when high-performance materials, showing a very high permeance and/or selectivity, are used. In this study, a series of steady-state separation performances experimentally obtained on CO_2_/CH_4_ mixtures with a zeolite membrane are compared to the predictions of a dedicated 1D approach, recently derived and validated through CFD simulations. Polarization effects are shown to generate a significant negative impact on the separation performances, both in terms of the productivity and separation efficiency. The 1D model predictions, based on pure gas permeance data and without any adjustable parameters, are in very good agreement with the experimental data. This fast and efficient modeling approach can easily be implemented in simulation or process synthesis programs for the rigorous evaluation of membrane gas separation processes, when high-performance materials are used.

## 1. Introduction

Membrane gas separation processes are considered today as a key technology for several important industrial applications, such as nitrogen-enriched air, hydrogen purification, natural and biogas upgrading, and VOC (Volatile organic compounds) recovery [1,2]. More recently, a great number of studies investigated the possibilities of membrane processes for carbon capture applications, with a strong emphasis on the development of high-performance materials (i.e., high permeance and/or high CO_2_/N_2_ selectivity) [3,4]. More generally, breakthrough performances for different gas mixtures have been reported in the last few years with a large number of inorganic and organic materials, offering attracting possibilities for different applications, compared to the current commercially available gas separation membranes [5,6].

From a process simulation point of view, membrane gas separations usually make use of a simple 1D approach, initially proposed by Weller and Steiner [7]. The set of assumptions includes steady-state, isothermal, isobaric, constant permeance and no-flux coupling conditions, together with the key hypothesis that the membrane is the only mass transfer resistance. The latter hypothesis globally holds for the membrane materials (almost exclusively dense skin polymers) that are currently used for industrial applications [8]. This simple simulation strategy has proven to be very effective for process design purposes [9]. Nevertheless, the negligible gas phase resistance hypothesis has to be logically reconsidered as soon as a membrane shows a very high permeance and/or a very high selectivity. In that event, a so-called concentration polarization effect, which is almost systematically considered for liquid membrane separation processes, has to be included in the simulation approach.

The peculiarities of gas separations logically have to be rigorously taken into account when the gas phase resistance in the boundary layer (i.e., where concentration polarization takes place) is computed [10,11]. Liquid separations classically postulate dilute solutions, with one predominant species (solvent), which can support a constant total molar concentration hypothesis in the liquid boundary layer, even for a high-molecular-weight solute [12,13]. A constant total transmembrane flux assumption is thus often proposed (i.e., total flux is close to solvent flux), which enables simple concentration polarization expressions to be used. Moreover, the dilution solute assumption enables simplified mass-transfer correlations to be used, where counter-diffusion effects in the boundary layer can be neglected [14]. The impact of this simplification, which corresponds to a negligible radial flow term in the gas boundary layer, has been shown to generate significant differences for mass flux computations [15]. Generally speaking, the ratio of transmembrane flux (*J*) over mass transfer coefficient (*k*) is shown to be the key factor that will affect the concentration polarization level (usually expressed as the concentration polarization factor, *c_m_*/*c_b_*, where *c_b_* is the bulk concentration of the more permeable species and *c_m_* its concentration in the gas phase at the membrane surface).

The previous conditions no longer apply to gas separations, where gas mixtures are rarely diluted (e.g., biogas contains about 40 vol% CO_2_ and 60 vol% CH_4_). Furthermore, volume fluxes are subject to variations due to phase compressibility effects. More generally, the use of simple mass transfer expressions can be questionable when correlations, based on impermeable wall conditions, are used together with a diluted mixture assumption. The interplay of species transmembrane fluxes and fluid velocity profiles close to the membrane interface could indeed generate unexpected effects on boundary-layer concentration profiles. This can potentially lead to incorrect computations, because one of the key targets for flux computation relies on the precise evaluation of the membrane interface solute concentration in the gas boundary layer (i.e., c_m_). The most rigorous simulation approach, in that case, is to perform CFD (Computational Fluid Dynamics) simulations, which enable 2D or 3D simultaneous resolutions of momentum, mass, and energy balances [16]. Surprisingly, very few studies have been reported where CFD is applied to membrane gas separations processes [17]. Additionally, a large portion of these studies are applied to H_2_/palladium separations, which show an infinite H_2_ selectivity (that is, a single-species transmembrane flux) [18,19,20]. Other publications explore the potential interest of complex membrane geometries in order to generate increased mass transfer coefficients, but with no focus on concentration polarization effects [21], or a focus on the enhancement of mass transfer coefficients by adding spacers in spiral membranes [22].

This situation stimulated the rigorous analysis of polarization effects with gas mixtures based on CFD (Computational Fluid Dynamics) simulations in a recent study [23]. A dedicated, generic, tailor-made 1D model has been extracted based on this exploratory study, which shows very good agreement with the CFD results. Nevertheless, the ability of this 1D approach to correctly predict experimental data when the concentration polarization is significant remains unexplored. This study intends to fill that gap through a systematic comparison of a series of experimental separation performance data obtained with a high-performance zeolite membrane to model predictions.

A high-permeance, high-selectivity zeolite membrane is used for different feed compositions, feed flowrates, and pressure ratios under steady-state separations. Retentate and permeate flowrates and compositions are measured, and mass balance is performed in order to check the data consistency. A systematic comparison between the experimental data and the 1D tailor-made model data, without any adjustable parameters (the only input is the pure gas permeance data), is then achieved. In the case of success, the simplified 1D approach will offer promising perspectives for process simulation packages over a broad range of conditions. The computing efforts of CFD simulations could then be circumvented, without losing prediction precision.

## 2. Materials and Methods

### 2.1. Experimental Section

The membrane separation performances are experimentally estimated using a commercially available tubular high-silica chabazite (Si-CHA) membrane from ZeoMem Sweden AB [24]. Pure silica chabazite is a zeolite framework composed of ellipsoidal cavities interconnected by narrow 8-membered oxygen ring windows. It has a three-dimensional pore network with a pore size of 3.8 × 3.8 Å [25]. The diameter of the pore openings is similar to the size of a methane molecule CH_4_ (3.8 Å), making chabazite membranes promising molecular sieves. For the separation of CO_2_/CH_4_ mixtures, membranes with the CHA framework type, in particular, aluminosilicate SSZ-13 or all-silica Si-CHA, show very high performance in terms of permeance and selectivity [26,27,28].

The studied membrane consists of a selective thin zeolite layer of 450 nm, deposited on an α-alumina tubular support which is about 8.5 cm long and has a 6 mm internal diameter. Thus, the effective membrane area is about 15.8 cm^2^. The membrane (and the support) was placed in a stainless-steel module (with gaskets) that was 10 cm long.

**Figure 1:**
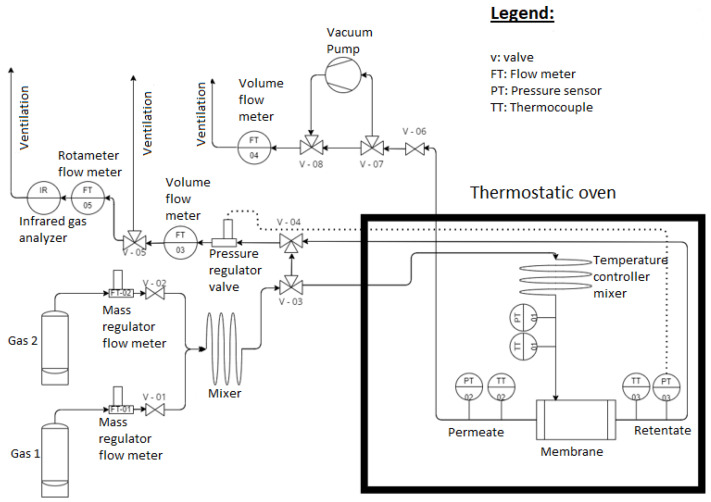
Schematic representation of the experimental setup used for gas separation measurements.

An in-house setup, shown in Figure 1, was designed and developed to carry out the gas permeation experiments. The setup can be operated in “dead-end” mode, where only the permeation flux of pure components is measured, while the retentate side is blocked. This enables pure gas permeance data to be obtained. It can also be operated in a classic mode under steady state, measuring both outlet permeate and retentate streams for the separation of carbon dioxide/methane CO_2_/CH_4_ gas mixtures.

The pure (>99.995%) carbon dioxide (CO_2_) and methane (CH_4_) were supplied from cylinders fitted with pressure gauges (Messer Group, Bad Soden, Germany). The inlet CO_2_ and CH_4_ flows were measured with mass flow meters/controllers (Bronkhorst High-Tech B.V., Ruulo, The Netherlands), while the outlet flows were measured with volumetric gas flow meters (Bios Defender, MesaLabs, Lakewood, OH, USA), both flowrates being converted to mol s^−1^. The membrane was fed in counter-current mode.

During the gas separation experiments, no sweep gas was used. The driving force (partial pressure difference) was generated by applying pressure to the upstream side, with the permeate side connected to the atmosphere or to a vacuum pump. The inlet and outlet pressures were constantly controlled with pressure sensors (S+S Regeltechnik GmbH, Serv’Instrumentation, Nürnberg, Germany). The pressure regulator valve was placed on the retentate side to maintain a constant upstream pressure of 5 or 10 bar(a). The membrane module was placed in the thermostatically controlled oven to ensure isothermal conditions (25 °C) during the experiments. An additional mixer was mounted inside the oven to ensure stable, constant temperature conditions. Three PT100-type thermocouples were placed in the gas stream on the feed, permeate, and retentate sides to continuously control the temperature. Three different CO_2_/CH_4_ inlet molar concentrations have been studied: 50/50, 70/30, and 90/10. Prior to each series of gas mixture separation experiments, the permeance of the pure gases was estimated at feed pressures of 5 and 10 bar(a). The composition of the retentate gas was measured continuously by infrared gas spectroscopy (e MGA3000 Multi-Gas Analyser, Hoddesdon, UK).

The transmembrane flux density *N_i_* of pure gases at steady state was calculated as follows:

(1) $$N_{i}=\frac{J_{i}}{S}$$

where *N_i_* is the molar permeation flux density in mol m^−2^ s^−1^, *J_i_* is the permeate molar flowrate in mol s^−1^, and *S* is the effective membrane area in m^2^. The membrane performance, in particular, the permeance, expressed as the permeation flux density *N_i_* divided by the driving force Δ*p_i_*, is estimated by Equation (2):

(2) $$P_{i}=\frac{N_{i}}{\Delta p_{i}}$$

where *P_i_* is the permeance of single gas *i*, initially expressed in mol m^−2^ s^−1^·Pa^−1^ (and subsequently converted to GPU (Gas Permeation Units) with 1 GPU = 3.348 × 10^−10^ mol m^−2^ s^−1^ Pa^−1^), and Δ*p_i_* is the partial pressure difference in Pa. The ideal membrane selectivity α_i/j_, which reflects the separation efficiency as the ratio of permeances of pure components *i* and *j*, is estimated by Equation (3):

(3) $$\alpha_{i/j}=\frac{P_{i}}{P_{j}}$$

For a gas mixture separation, the concept of stage cut *θ* is of major importance and expresses the ratio of the molar flux passing through the membrane compared to the feed flux:

(4) $$\theta =\frac{J_{perm}}{J_{feed}}$$

where *J_perm_* and *J_feed_* are the molar flowrates in mol s^−1^ of the permeate and feed streams, respectively.

### 2.2. Modeling Approaches

The aim of this study is the modeling of the concentration polarization phenomenon and its influence on membrane separation processes in the case of high-performance inorganic membranes. In order reach this objective, a comparison of two models is carried out. The first one is a classical 1D model that does not take into account the concentration polarization phenomenon, and the second one is an improved model that considers this phenomenon under steady-state conditions, which is the case for most industrial applications.

In both cases, the gas mixture is considered to be fed into the lumen side of the fiber, which represents the configuration of the experimental setup, and the shell side (permeate) is considered to be under perfect vacuum conditions, which is the case that maximizes concentration polarization (i.e., maximal transmembrane flux). Membrane properties such as permeance and selectivity are considered as constant. The gas mixture is assumed to be an ideal gas, which corresponds to the operating conditions used in this study (moderate feed pressure and a temperature of 25 °C), and the gas properties such as mass diffusivity and dynamic viscosity are considered constant throughout the process.

The geometry for both models corresponds to the dimensions of the actual membrane used in the experiments, i.e., 8.4 cm effective length with an internal diameter of 6 mm.

#### 2.2.1. Classical 1D Model

The classical 1D modeling approach is a simplified method for predicting the performance of a membrane gas separation process, first proposed by Weller and Steiner [7]. It is based on the assumption that the membrane is the only resistance to mass transfer. It assumes a uniform concentration on both sides of the membrane and neglects concentration polarization. In addition, each compartment (the permeate side and the retentate side) is generally considered to be isobaric and isothermal. Further developments have included the effects of heat transfer and pressure drop [29].

In most industrial applications of membrane gas separation, modules with polymeric membranes are used due to their ease of production capacity and low cost. For such membranes, which are characterized by moderate permeances and selectivity, concentration polarization is less likely to be important. For this reason, classical 1D models such as [9] are widely used for the design of membrane separation processes. In addition, they can be used in more complex algorithms to predict the perfect architecture and/or operating conditions for a membrane separation process [9,29].

In this study, the classical 1D model is developed with the following assumptions:

- Negligible pressure drop in each side of the membrane;
- Isothermal process;
- Gas mixtures follow the ideal gas law;
- High Péclet number (negligible axial diffusion);
- Constant gas permeance;
- Negligible coupling effects;
- Vacuum conditions at the permeate.

**Figure 2:**
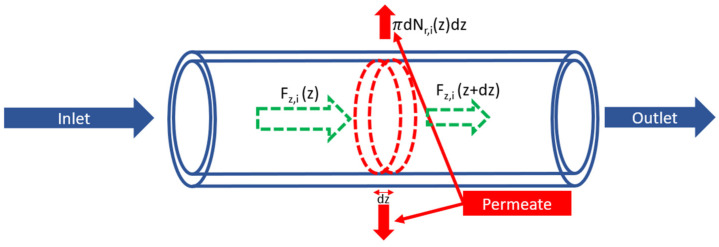
Mole balance over an elementary volume (dashed) in the membrane tube.

The mass balance of an elementary volume of the membrane (Figure 2) gives:

(5) $$\frac{{dF}_{z,i}(z)\;}{dz}=\pi dN_{r,i}\left(z\right)$$

where *i* represents the specie in the mixture, *z* is the axial position through the membrane in *m*, *d* is the fiber diameter in *m*, *F_z_* is the local lumen-side molar flowrate in the axial direction in mol s^−1^, and *N_r_* represents the local permeation flux density (radial) in mol m^−2^ s^−1^.

Considering the hypothesis of uniform concentration on each side of the membrane used in this type of model, and given that perfect vacuum is applied at the permeate side, the permeation flux density is calculated as per Equation (6):

(6) $$N_{r,i}\left(z\right)=P_{i}\left(p_{i,int}\left(z\right)-p_{i,ext}\left(z\right)\right)=P_{i}p_{i,int}\left(z\right)=P_{i}px_{i}(z)$$

where *P_i_* is the permeance in mol m^−2^ s^−1^ Pa^−1^, *p_i_* is the partial pressure in Pa, *p* represents the pressure of the flow in *Pa*, *x_i_* is the mole fraction of a specie in the mixture, and *int* and *ext* correspond to the lumen (retentate) and shell (permeate) sides, respectively.

By solving Equations (5) and (6), axial fluxes are determined for each species, then mass balances and the ideal gas law are used to derive concentrations and mole fractions for each species.

#### 2.2.2. Improved 1D Model

The experimentally used membrane presents high separation performances compared to the usually employed polymeric membranes; moreover, inorganic membranes are provided with a larger internal diameter and smaller length. All these characteristics favor the occurrence of the concentration polarization phenomenon. Thus, the need to develop a model that takes into account this phenomenon in order to study in depth the impact of these new materials on membrane gas separation processes and their opportunity to be more favored in industrial applications.

Such models have been developed and improved over the years, starting from [10] who predicted the permeation rate from which concentration polarization becomes important; a 1000 GPU permeance level and a selectivity larger than 2 are considered as the limit above which polarization plays a significant role. In a simulation study, He et al. suggested that polarization effects are significant as soon as the permeance exceeds 100 GPU [15]. Later on, Mourgues and Sanchez [30] set the limit of a permeance of 1000 GPU and a membrane selectivity of 100 as the limit at which concentration polarization becomes significant. Recently, several models have been developed to include other phenomena that can interplay with concentration polarization, such as the Joule–Thompson effect, competitive sorption, etc., in order to study the influence of each phenomenon on the process [31,32]. All these models predict global results at the outlets of the module (permeate side and retentate side), but the absence of local results at each axial position of the fiber makes the full understanding of the concentration polarization more complicated. Therefore, a generic model is detailed hereafter that predicts the separation characteristics while taking into account the concentration polarization locally along a membrane fiber, without any simplifying assumption.

**Figure 3:**
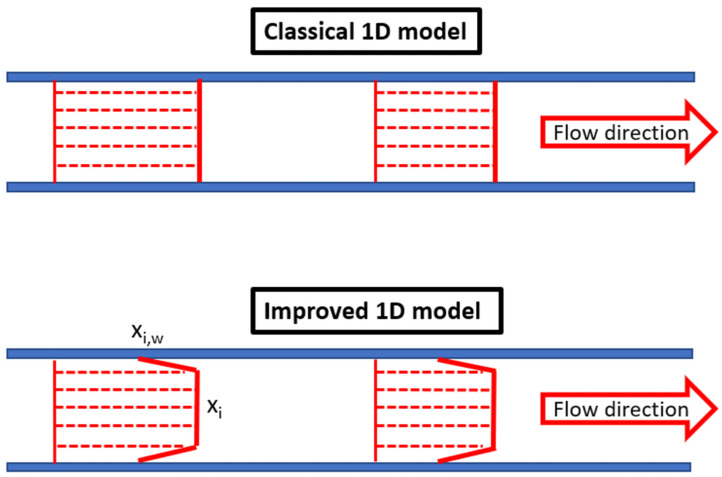
Schematic representation of the two modeling approaches for the more permeable species.

The main purpose of this model is to calculate two different molar fractions for every axial position. The first is the bulk molar fraction, i.e., the mixing-cup molar fraction, and the near-wall molar fraction, which represents the molar fraction at the interface gas separation layer of the membrane as shown in Figure 3. This method, by calculating the near-wall molar fraction, considers the limitation in the gas phase by replacing the bulk partial pressure of Equation (2) by the near-wall molar fraction. For the more permeable species, the driving force is less than that of the classical 1D model, while for the less permeable ones, the driving force is higher.

Considering the vacuum conditions at the permeate side, the mass balance over an elementary volume inside the membrane tube is written as:

(7) $$N_{r,i}\left(z\right)=k_{i}^{app}\left(z\right)c\left(x_{i}\left(z\right)-x_{i,w}(z)\right)\;{=P}_{i}\left(p_{i,w}\left(z\right)-p_{i,ext}\left(z\right)\right)=P_{i}p_{i,w}\left(z\right)=\;P_{i}px_{i,w}(z)$$

where $k_{i}^{app}$ refers to the convective mass transfer coefficient of the species *i* at the lumen side in m s^−1^, *c* is the molar concentration of the mixture in mol m^−3^, and the subscript *w* represents wall, which means the property at the solid–gas interface.

Correlations for the convective mass transfer coefficient are developed for flows in pipes under the assumption of an extremely diluted mixture. Because of that, a correlation developed for the heat transfer of a simultaneously developing flow in a round pipe by Shome and Jensen [33] is used:

(8) $${Sh}^{dil}\left(z\right)=\frac{k^{dil}\left(z\right)\;d}{D_{gas}}\left\{\begin{array}{c} =\left[3.6568+0.2249\;{Gz}^{0.4956}exp\left(\frac{-55.9857}{Gz}\right)\right]{\left[1+98.42\left(1+{Gz}^{0.24}\right)\tanh\left(\frac{0.93}{{Sh}_{w}}\right)\right]}^{0.03}\\ \times {\left[1+0.004\left(1+\tanh\left(\frac{61.74}{{Sh}_{w}}\right)\right)\;\frac{Gz}{Sc}\right]}^{0.12}\;\;\;\;\mathrm{if}\;Gz\leq 10^{3}\\ =\left(-0.3856+1.022\;{Gz}^{0.3366}\right){\left[1+98.42\left(1+{Gz}^{0.24}\right)\tanh\left(\frac{0.93}{{Sh}_{w}}\right)\right]}^{0.03}\;\;\;\;\;\;\;\;\;\;\;\;\;\;\;\;\;\;\;\;\;\;\;\;\;\;\;\;\;\;\\ \times {\left[1+0.004\left(1+\tanh\left(\frac{61.74}{{Sh}_{w}}\right)\right)\;\frac{Gz}{Sc}\right]}^{0.12}\;\;\;\mathrm{if}\;Gz\geq 10^{3} \end{array}\right.$$

where *Sh^dil^* is the Sherwood number for an infinitely diluted solution, *Sc* represents the Schmidt number of the mixture, and *Gz* is the local Graetz number.

This correlation is then corrected twice: at the beginning to consider the transmembrane flux, i.e., diffusion-induced advection in the radial direction as in Equation (9) and then, to take into account the real situation of concentrated mixtures, Equation (10), which is the case for membrane gas separation applications [34]:

(9) $$N_{r,i}\left(z\right)=k_{i}^{app}\left(z\right)\;c\left(x_{i}\left(z\right)-x_{i,w}\left(z\right)\right)=x_{i,w}\left(z\right)\sum_{j=1}^{2}N_{r,j}(z)+k^{dil}\left(z\right)\;\;\frac{\ln\left[1+R_{i}(z)\right]}{R_{i}(z)}c\left(x_{i}\left(z\right)-x_{i,w}\left(z\right)\right)$$

(10) $$R_{i}\left(z\right)=\frac{\left(x_{i,w}\left(z\right)-x_{i}\left(z\right)\right)\sum_{j=1}^{2}N_{r,j}\left(z\right)}{N_{r,i}\left(z\right)-x_{i,w}\left(z\right)\sum_{j=1}^{2}N_{r,j}\left(z\right)}$$

By solving the differential Equation (5) with Equation (7) using Correlation (8) and its Corrections (9) and (10), it is possible to simultaneously calculate the bulk and wall molar fractions and the axial flux along the fiber length. Then, all remaining properties can be obtained from mass balance equations. This model was compared and validated locally for a wide range of operating conditions with a complete 2D CFD model [23].

## 3. Results

### 3.1. Local Comparison of the Different Modeling Approaches

This section presents a comparison of the stage-cut and retentate molar fractions of carbon dioxide’s axial profile using the following operating conditions, which represent one of the experimental conditions tested. An equimolar mixture of carbon dioxide and methane is fed to the lumen side of the module at a flux of 2 NL min^−1^ under 5 bar pressure at 25 °C on the retentate side, while the permeate side is under vacuum. The permeances are 9120 GPU and 50 GPU for carbon dioxide and methane, respectively, giving a membrane selectivity of 182.

**Figure 4:**
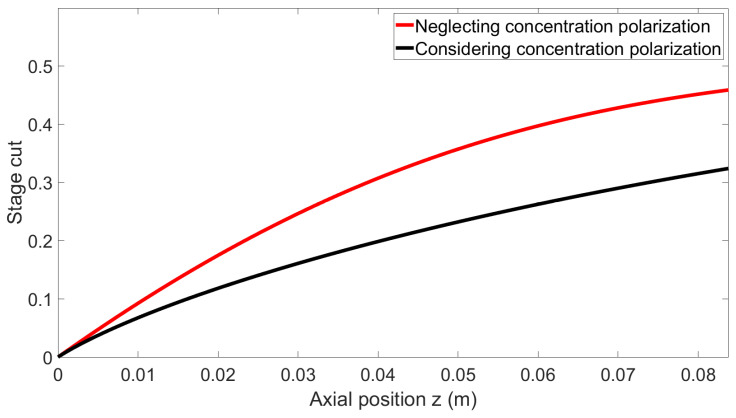
Local stage cut as a function of the axial position for both models.

**Figure 5:**
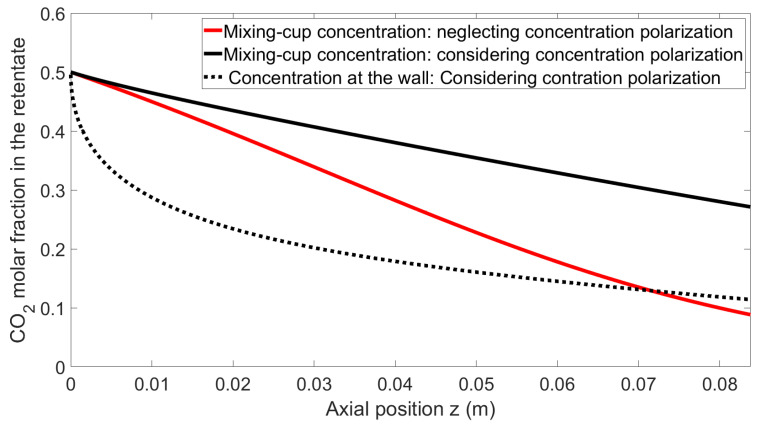
Local CO_2_ molar fraction at the lumen side (retentate) as a function of z for both models.

As shown in Figure 4, neglecting the phenomenon of concentration polarization for high-performance membranes leads to an overestimation of membrane productivity (i.e., permeate flowrate). Meanwhile, Figure 5 shows that in addition to productivity (permeate flowrate) error, the purity of the retentate side, which is the most important specification for biomethane purification, is also largely overestimated.

Finally, the molar fraction at the wall is significantly lower than that of the bulk, clearly demonstrating the importance of concentration polarization for these operating conditions.

This first simulation set shows that, based on a systematic model comparison, the impact of concentration polarization is quantitatively very significant, both in terms of the productivity (permeate flowrate) and separation performances (outlet purity) for the key characteristics of the system selected for experiments. This validates the choice of the membrane, mixture, and operating conditions for a study dedicated to concentration polarization effects. The key remaining question regarding the possibility of the model to correctly predict the experimental data is detailed hereafter.

### 3.2. Experimental Validation of the Model

In this section, the comparison of the numerical and experimental results for different concentrations of the CO_2_/CH_4_ mixture is presented in two different cases: one using a vacuum pump on the permeate side and one bypassing the pump in order to have atmospheric pressure on the permeate side.

For the case of the permeate side at atmospheric pressure, an assumption of perfect mixing on this side is added to both models, considering and neglecting concentration polarization. The measured composition on the permeate side is used to calculate the driving force of the process. Equations (6) and (7) are then rewritten as Equations (11) and (12), respectively:

(11) $$N_{r,i}\left(z\right)=P_{i}\left(p_{i,int}\left(z\right)-p_{i,ext}\left(z\right)\right)=P_{i\;}\left(p_{i,int}\left(z\right)-p_{atm}y_{i}\right)$$

where *p_atm_* is the atmospheric pressure and *y_i_* is the measured molar fraction of the species *i* on the permeate side.

(12) $$N_{r,i}\left(z\right)=k_{i}^{app}\left(z\right)c\left(x_{i}\left(z\right)-x_{i,w}(z)\right)\;{=P}_{i}\left(p_{i,w}\left(z\right)-p_{i,ext}\left(z\right)\right)=\;P_{i}\left(px_{i,w}\left(z\right)-p_{atm}y_{i}\right)$$

**Figure 6:**
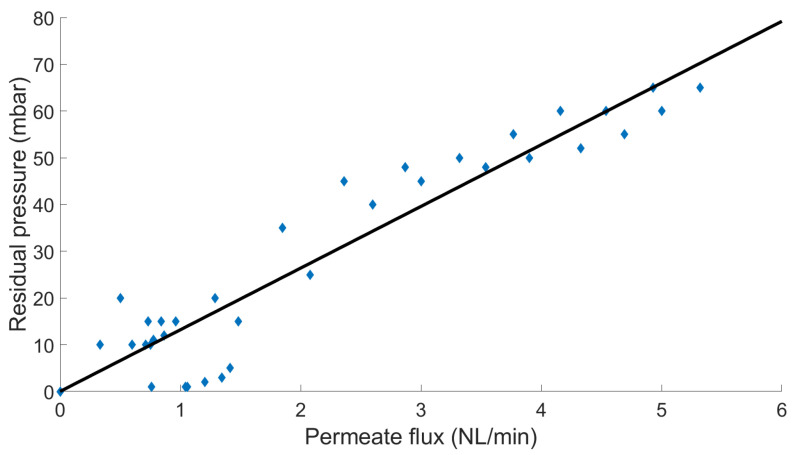
Residual pressure on the permeate side of the membrane as a function of the transmembrane flux, the blue dots represents the measured pressure on the permeate side, the black line is the deduced pump curve.

In case of using a vacuum pump on the permeate side of the membrane, a residual pressure should always be taken into account, which corresponds to the pump capacity as a function of the effective transmembrane flux. For the pump, which is experimentally used, the residual permeate pressure is a linear function of the permeation flux as shown in Figure 6.

The effect of this residual pressure is studied by comparing the numerical results of two cases. The first case follows the previous procedures to consider this residual pressure, then it is compared with the results of simulations considering perfect vacuum conditions. For the operating conditions investigated experimentally with mixture, the measured residual pressure is below 10 mbar. In this case, the difference between the two approaches was found to produce a maximum relative error below 1%, so for the rest of this study, in the case of using the vacuum conditions, a perfect vacuum is assumed in the modeling procedure: Equations (6) and (7) are thus solved accordingly.

#### Comparison between Experimental and Simulation Results

Before each gas mixture experiment, the pure gas permeances were measured. These permeances and selectivities are used as inputs for both models to produce the numerical simulation results. For all concentrations of the CO_2_/CH_4_ mixture, feed fluxes ranging from 1 to 5 NL min^−1^ were tested (1, 1.5, 2, 3, 4, and 5, respectively), with two different pressure levels on the retentate side (5 and 10 bar), and two pressure conditions on the permeate side (vacuum conditions and atmospheric pressure) at a constant temperature of 25 °C. It is expected that this range of operating conditions provides a large enough set of separation performances in order to correctly evaluate the model prediction possibilities.

For an equimolar mixture at the inlet, the measured methane permeance, which is experimentally shown to be independent of pressure, is 50 GPU. The CO_2_ permeance is pressure-dependent and decreases with increasing pressure, as reported by several studies with zeolite membranes [35,36]. The effective permeances are 9120 and 8316 GPU for 5 and 10 bar, respectively, giving a membrane selectivity of 182 for 5 bar and 166 for 10 bar.

Another feed concentration was tested, with 70 vol% CO_2_ and 30% CH_4_ on the feed side of the module. In this case, the pure gas permeances are 30 GPU for methane and 4500 and 4250 GPU for carbon dioxide at 5 and 10 bar, respectively, resulting in a membrane selectivity of 150 at 5 bar and 142 at 10 bar. It should be noted here that the pure gas permeances were observed to decrease over time, probably due to the presence of some trace water vapor in gas cylinders which may be adsorbed to the membrane material without being desorbed. The downgrade of these performances was slow enough to consider a constant CO_2_ permeance during each experiment session, by measuring it before and after each session.

**Figure 7:**
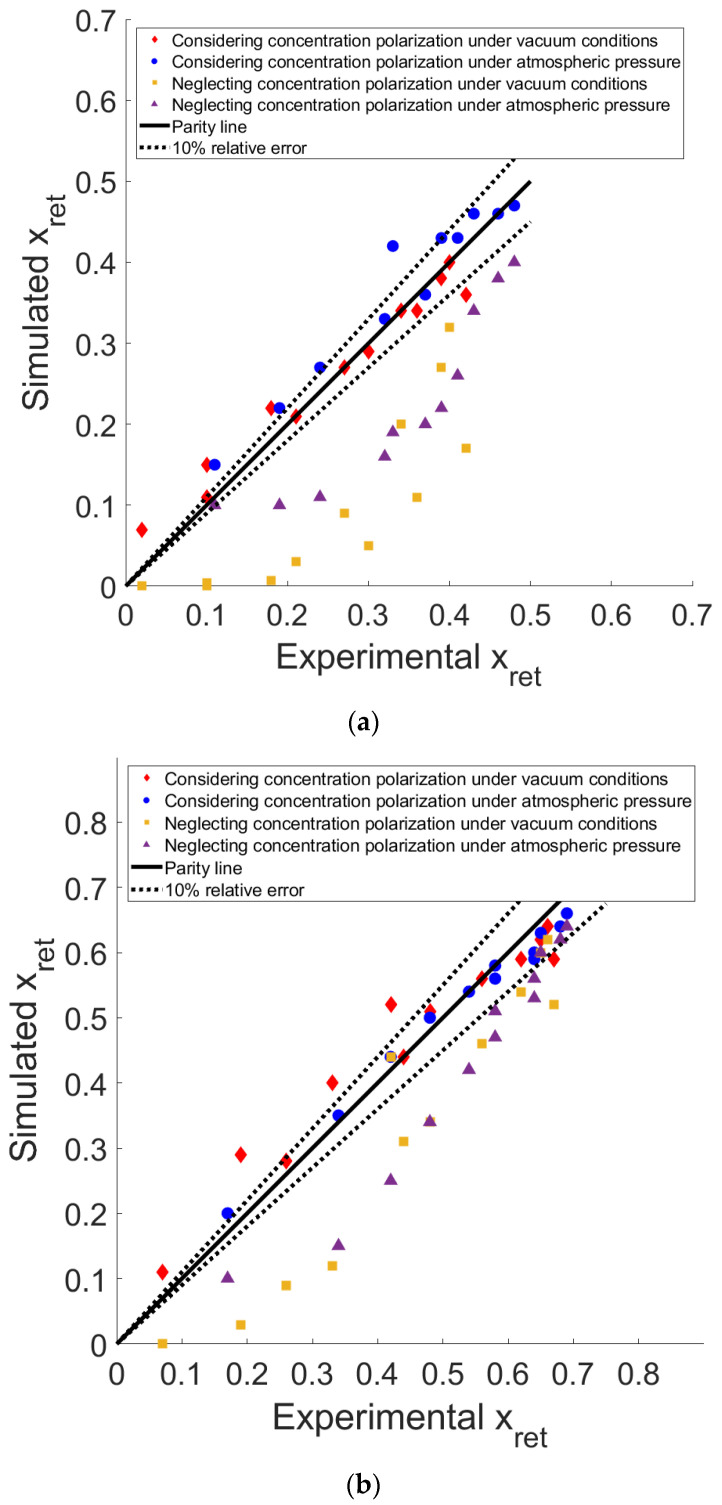
CO_2_ outlet mole fraction on the retentate side (x_ret_): comparison of predictions for both models with experimental results, (**a**) equimolar feed mixture, (**b**) 70% CO_2_ in the feed mixture.

The comparison between the simulated and experimental results is shown in Figure 7a for an equimolar feed mixture and Figure 7b for an input of 70% of CO_2_ and 30% CH_4_.

As shown in Figure 7a,b, the numerical predictions of x_ret_ (at the outlet of the membrane fiber), the outlet CO_2_ molar fraction (i.e., at the end of the module), using the improved model that accounts for concentration polarization are within a 10% relative error of the experimental results for most of the operating conditions. The points that correspond to low x_ret_ values are the points with the lowest feed flow rates. These low fluxes are difficult to be precisely measured and controlled and have led to the highest relative error ranges. Meanwhile, for the classical model that does not consider this phenomenon, the prediction of the resulting composition is an overestimation in terms of purity, especially for lower x_ret_ values at the end of the module, which refers to the lower feed fluxes. At higher feed fluxes, the results of the two models are closer, and this can be explained by the reduction in the residence time in the module; in that case, the change of the composition between the inlet and the outlet of the module is limited because of the higher fluxes (small stage-cut conditions).

A feed composition of 90% CO_2_ and 10% CH_4_ was also tested. As with the previous composition, pure gas permeances were measured prior to testing with mixtures. The CO_2_ permeance obtained was 4260 GPU at 5 bar and 3834 GPU at 10 bar, while for CH_4_, it was 30 GPU for both pressures. This gives a membrane selectivity of 142 and 127.8 for 5 and 10 bar, respectively.

**Figure 8:**
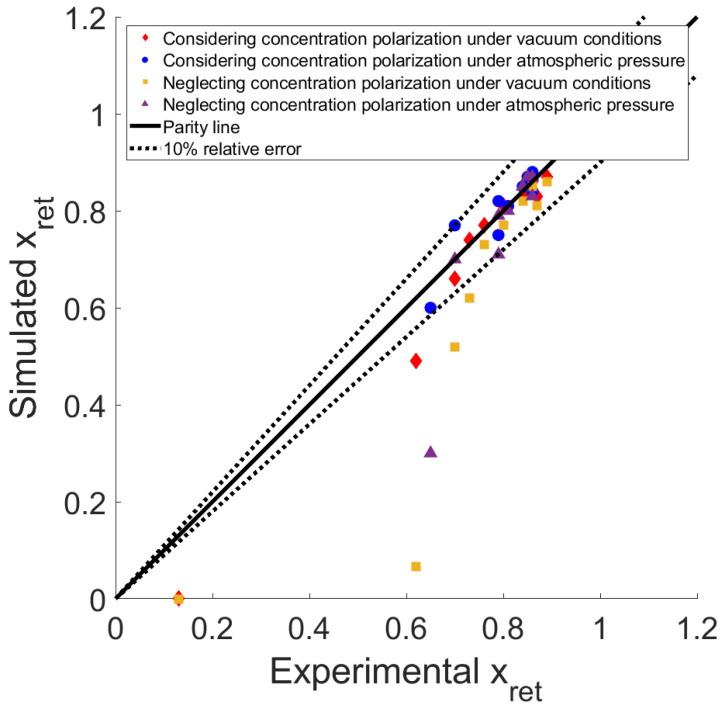
CO_2_ outlet mole fraction on the retentate side (x_ret_): comparison of predictions for both models with experimental results. Feed composition: 90% CO_2_ and 10% CH_4_.

Figure 8 shows that at low fluxes, the difference between the models is large, but at higher fluxes, the two models predict the final composition within 10% of the relative error compared to the experimental data. This can again be explained by the decrease in the residence time in the module and/or by the reduced influence of concentration polarization under these conditions.

## 4. Discussion

In the present study, two models for predicting the performances of membrane gas separation modules have been presented. The first model is based on a classical approach that considers the uniform concentration for each axial position throughout the membrane, thus neglecting the concentration polarization phenomenon. The second model calculates the wall concentration along the bulk concentration for each axial position to account for this phenomenon.

The predictions of both models are compared with experimental data; the comparison clearly demonstrates the fact that with the use of new inorganic membranes presenting higher permeances and selectivities, the effect of concentration polarization should be considered, which agrees with the performances presented in the literature indicating the limit of 1000 GPU for the most permeable specie with a membrane selectivity of 100 [30] for neglecting this phenomenon. The lowest carbon dioxide permeance measured in this study was 3834 GPU with a selectivity of 127.8, which is above the mentioned limit.

The comparison also validates the model considering concentration polarization, as the relative errors between the predicted numerical results and the measured experimental results are less than 10% for all concentrations tested. It is important to point out that the coupling effects of constant permeance and no flux have been taken as assumptions for the simulations. The very good agreement between the 1D model and experimental results suggests that permeance variability and CO_2_/CH_4_ interactions do not significantly impact the species fluxes. This situation is interesting for simulation purposes, but it is not systematic at all. For membrane systems which show one of these complicating effects, a more refined simulation approach is required, but it can be implemented in the model reported above.

## 5. Conclusions

Concentration polarization effects are systematically taken into account for liquid membrane separations but are most often neglected for membrane gas separations. A limited number of studies up to now have addressed the prediction possibilities of simulation approaches when polarization effects are significant. CFD is expected to offer the best simulation performances in that case, but it requires significant computing efforts. A recent strategy suggests making use of a dedicated, fast, and efficient 1D-type approach. It has been shown to generate separation performances predictions very close to CFD results. This study has shown that concentration polarization effects produce significant performance decreases when a zeolite membrane is used for biogas upgrading applications. Interestingly, the effective separation performances can be predicted within a 10% max error limit thanks to the simple, dedicated 1D model. This approach does not require any adjustable parameters, but simply the pure gas permeance data, in combination with the system properties included in the mass balances and transmembrane flux expressions. The high retentate bulk concentrations and counter-diffusion effects are included in the approach, which provides a generic application potential. Moreover, the modeling approach can easily be extended to multicomponent mixtures or systems where variable permeance and/or coupling effects play a significant role.

Besides the interest of the 1D model to improve gas separation process simulations with high-performance membranes, its simplicity and efficiency offers promising perspectives for process synthesis studies (Process System Engineering), where heavy CFD simulations cannot be implemented in optimization algorithms unless high-performance computing means are used. Similarly, parametric sensitivity studies can be easily achieved with this model in order to quickly address the key question of the significant or negligible impact of concentration polarization on separation performances. More specifically, this study has shown that the usual 1000 GPU permeance and 100 selectivity boundaries, which are often recommended for polarization effects to be taken into account, have to be reconsidered. No systematic set of data can, in fact, enable the prediction of the importance of concentration polarization; depending on the membrane characteristics (permeance and selectivity), module geometry, operating conditions (pressure ratio, stage cut), feed mixture composition, and specifications, a classical 1D Weller and Steiner simulation may be appropriate or not. This information completely changes the simulation strategy and efforts. The answer can be obtained with a simple standalone computer program that should be of great help, be it for experimental data interpretation or process design studies.

## Data Availability

The raw data supporting the conclusions of this article will be made available by the authors on request.

## Nomenclature

c	Molar concentration (mol m^−3^)
d	Membrane diameter (m)
D_gas_	Gas diffusion coefficient (m^2^ s^−1^)
F	Axial flow rate (mol s^−1^)
Gz	Graetz number (-)
J	Transmembrane flow rate (mol s^−1^)
k	Mass transfer coefficient (m s^−1^)
N	Total molar flux density (mol m^−2^ s^−1^)
N_r_	Local molar flux density (mol m^−2^ s^−1^)
p	Pressure (Pa)
P	Permeance (mol m^−2^ s^−1^ Pa^−1^)
S	Surface (m^2^)
Sc	Schmidt number (-)
Sh	Sherwood number (-)
x	Mole fraction in the lumen side (-)
y	Mole fraction in the permeate side (-)
z	Axial position (m)

**Greek Letters**	
α	Membrane selectivity (-)
θ	Module stage cut (-)

**Subscripts**	
b	Bulk
i	Species in the mixture
m	Membrane interface
r	Radial direction
ret	Retentate
w	Near wall
z	Axial direction

**Superscripts**	
app	Apparent
dil	Dilute
